# Cannulation via the external jugular vein——An alternative to conventional peripherally inserted central catheterisation for paediatric patients

**DOI:** 10.1186/s12887-023-04403-5

**Published:** 2023-11-18

**Authors:** Ping Zhang, Miao Jia, Wan-yuan Li, Juan Li, Jin-lei Niu, Hong Ding, Wang-mei Zhou

**Affiliations:** 1 Division of Orthopaedics and Traumatology, Department of Orthopaedics, Nanfang Hospital, Southern Medical University, Guangzhou, 510515 China; 2 Open Fracture and Limb Reconstruction Nursing Professional Committee, Guangdong Nursing Association, Guangzhou, 510170 China; 3 Department of Pediatrics, Nanfang Hospital, Southern Medical University, Guangzhou, 510515 China; 4 Department of Anesthesiology, Nanfang Hospital, Southern Medical University, Guangzhou, 510515 China; 5 Department of Emergency, Nanfang Hospital, Southern Medical University, Guangzhou, 510515 China

**Keywords:** Peripherally insertion central catheterization, External jugular vein, Modified cannulation, General Anaesthesia

## Abstract

This study aimed to describe a peripherally inserted central catheterisation (PICC) for paediatric patients with inaccessible access and a high risk of general anaesthesia (GA). Methods: This was a retrospective observational study involving all paediatric inpatients who performed the PICC via an EJV approach without GA between September 2014 and September 2021 in a provincial key clinical speciality. Results: A total of 290 EJV line placement attempts were performed, and 29 were excluded due to missing placement results, resulting in a sample size of 261. The anatomical localisation, punctures, and catheterisation success rates for this practice were 100%, 100%, and 90.04%, respectively. The placement success rate in children younger than one year was 93.75% (45/48). The median line duration of use was 19 days, with a median length of catheter insertion of 13 cm. The most common complications were catheter malposition (n = 20) and dislodgement (n = 7). Conclusion: The PICC via an EJV approach without GA is a feasible and safe practice with acceptable success and complication rates, and low costs. It might be an attractive alternative for obtaining central vascular access for paediatric patients.

**Supplementary Information:**

The online version contains supplementary material available at 10.1186/s12887-023-04403-5.

## What is known

The PICC procedure in children can often be challenging, and most children need to be sedated or under GA during the cannulation. However, GA is associated with complications and prominent risks. An alternative to conventional PICC is needed for paediatric patients with difficult venous access and a high risk of GA.

## What is new

This study reported a 7-year paediatric practice in our department that the experienced nurse used the central venous catheters to perform the PICC via the EJV approach for children without GA. It had a pleasing success rate in anatomical localisation (100%), punctures (100%) and catheterisation (90.04%), and with an acceptable rate of complications (11.88%), and might be an attractive alternative to conventional PICC for paediatric patients with inaccessible access and a high risk of GA.

## Introduction

Although a variety of techniques have been used to improve the procedure, peripherally insertion central catheterisation (PICC) in children can often be challenging [1]. This is mainly due to factors such as small vein size, hemodynamic instability, and a higher frequency of anatomical variations in the paediatric population [2]. Lack of cooperation is also an important cause of placement failure in children, especially in infants and younger children [3]. Most children need to be sedated during the procedure in order to optimize the positioning of the insertion site, to keep it in place and to reduce patient discomfort [4]. Some younger children require general anaesthesia (GA) [5]. However, GA is associated with complications [6], including early postoperative apnoea, respiratory depression, shock and cardiac arrest, and has the potential to affect the long-term neurodevelopment of children [7]. Prominent risks are associated with GA exposure in children with complex conditions, like chronic respiratory diseases [8] and coagulopathy [9].

The issue emerges. What should clinicians do when the risk of GA is incredibly high whilst the PICC is particularly necessary? To make the puzzle even harder, what could we do when routine access (e.g. basilic or cephalic veins) is inaccessible? The second cannulation site of choice which does not require GA is needed. The external jugular vein (EJV) might be an attractive alternative. It is a superficial and large peripheral vein, commonly visible and palpable, and can serve as an alternative access for PICC [10, 11]. The EJV can be easily localised even if children lack cooperation, or even cry and fuss. Not only that, the crying and fussing of the children are beneficial to the insertion because the EJV would be more visible when the children are crying and fussing, which leads to a pleasure outcome that it is not essential to perform this procedure under GA if appropriate constraints are provided [12]. New challenges arise. If we select a conventional peripheral inserted central catheter (common size: 50–70 cm) and an EJV to be cannulated, there will be a long external catheter, resulting in the necessary catheter trimming to obtain optimal catheter positioning [13, 14]. However, this action may lead to an increased risk of deep vein thrombosis [15]. Is there a shorter, suitable catheter for this operation? A central venous catheter (CVC, common size: 5-30 cm) may be the key to the lock. Our department’s preferred practice is to use a CVC to conduct the PICC without GA via the EJV approach for children with inaccessible access and a high risk of GA. As a Promotion of Appropriate Health Technology of Guangdong province, this practice has been implemented in our institute for 7 years. The study aimed to describe this practice and evaluate its feasibility and safety in a paediatric population.

## Materials and methods

### Study design, setting and participants

This was a retrospective observational study involving all inpatients with a CVC insertion via the EJV access without GA between September 2014 and September 2021 in the Department of Paediatrics at Nanfang Hospital, Southern Medical University. The department is a provincial key clinical speciality for the treatment of paediatric haematological diseases, and the EJV peripheral inserted central venous access is judged one of several routine access techniques that is standard practice for the study site. The study was approved by the ethics committee of Nanfang Hospital, Southern Medical University (No. NFEC-2022-511) and a waiver of consent was granted. Consolidated criteria for reporting observational studies (STROBE) were followed to ensure standardised reporting [16].

The indication for the insertion in our department included (1) patients with difficult venous access (2) patients at high risk for PICC under GA (3) patients whose parents do not consent to perform PICC under GA and (4) a temporary alternative for paediatric patients with coagulopathy until a conventional PICC can be performed. Contraindications included (1) infections, burns, or injuries near the neck or the EJV site (2) damaged or thrombosed vessels caused by previous catheter insertions or repeated attempts (3) mass in the neck causing compression symptoms or enlarged lymph nodes in the neck and (4) tracheotomy or other neck surgery for obstruction of superior vena cava return. All PICCs via the EJV approach performed in the department during the 7 years were included in the current study, except for those with missing placement results.

### Procedure

**Fig. 1:**
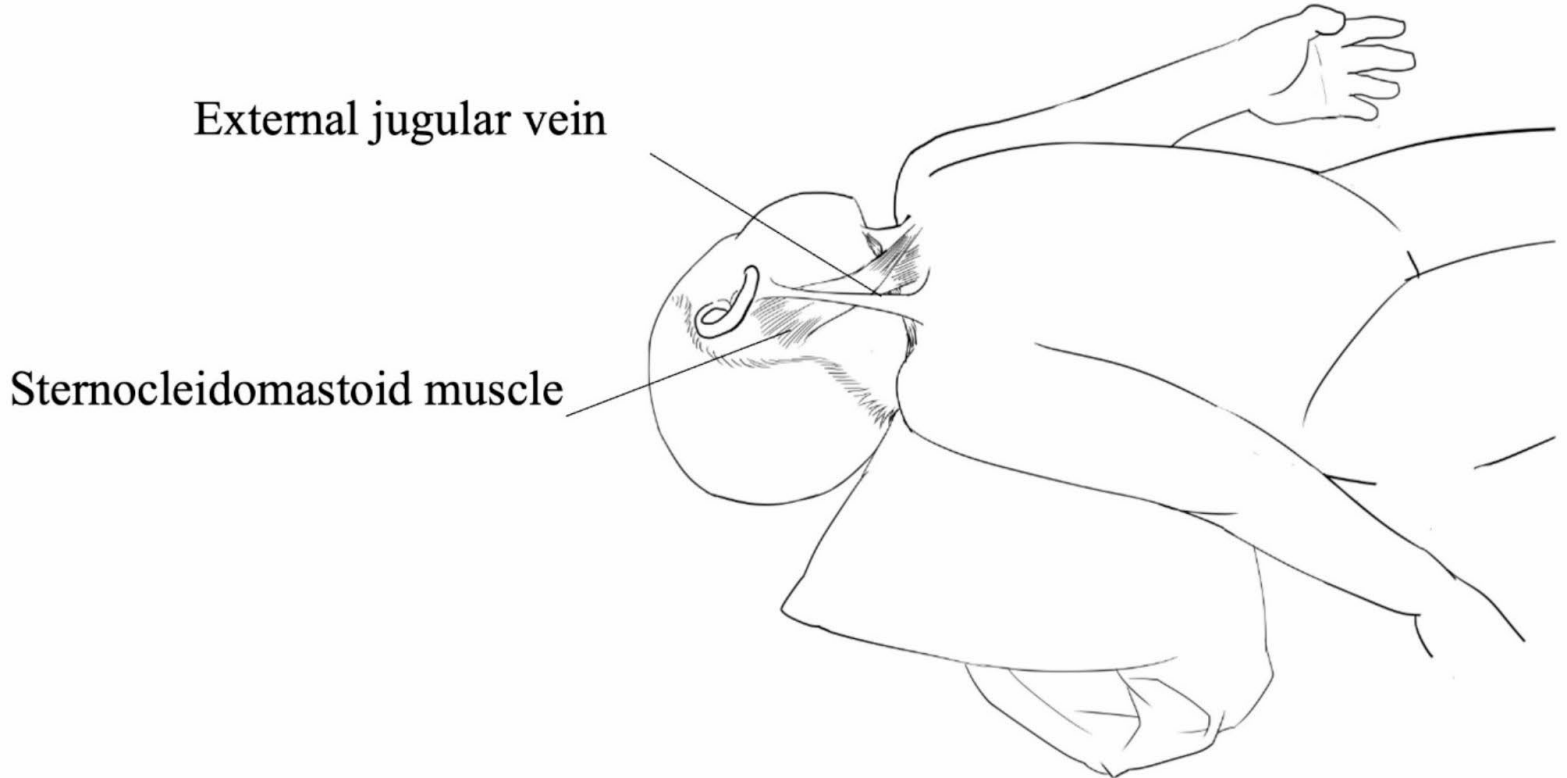
The body position of the child in the practice

All placements were performed using external anatomical landmarks by a specially trained PICC nurse and two registered nurses (RN) assisting. The PICC nurse who performed the EJV placements had first attempted this practice in early 2014 after five years of experience with conventional placement. The child lay on the bed with a pillow under the shoulders, one nurse wrapped the child’s body in a soft blanket, and the other nurse gently tilted the child’s head opposite the puncture site. Light pressure was applied above the clavicle to help visualise the selected EJV. The body position of the child is shown in Fig. 1. Either one one-lumen or two-lumen Fornia® (Royal Fonia Medical Equipment Co., Ltd, Zhuhai, China) disposable central venous catheter kit was implanted. The size of the lumen was 4–5 Fr (Chinese catheter gauge, 13-20 cm), depending on the age and vascular status of the paediatric patients. All catheters consisted of X-ray impervious medical grade polyurethane. Chest radiographs were obtained after the procedure to confirm the catheter tip position.

### Data collection and analysis

Data were captured from electronic patient records and included patients’ demographics (age, gender, height, weight and disease diagnosis), the total number of attempts, the success rate of punctures, the site of insertion, the success rate of placement, length of catheter insertion, duration of use, indications for line removal, and complications. Duration of use was viewed as the sum of follow-up days from the time of catheter insertion to the removal, patient death, and patient transfer to other centres or discharge to home with PICCs and failed to visit for follow-up. Complications were further divided into immediate and delayed complications (see Appendix Table 1).

**Table 1:** Baseline characteristics of the included patients (N = 261)

Characteristic	
Male sex, n (%)	169 (64.80)
Age in mouths, n (%)	
1–12 13–60 61–120 121–204	54 (20.69) 81 (31.03) 85 (32.57) 41 (15.71)
Weight in kg, Median (IQR)	16.75 (12.40, 23.57)
Height in cm, Median (IQR) (n = 237)	113 (91, 130)
Diagnoses, n (%)	
Leukaemia Pulmonary infection Hemophagocytic syndrome Severe pneumonia Graft-versus-host disease Others^a^	146 (55.94) 15 (5.75) 7 (2.68) 6 (2.30) 6 (2.30) 81 (31.03)

^a^ Diagnoses numbering no more than six cases were categorised into this group

Statistical analysis was performed using SPSS 20.0 software (IBM Corp, Chicago, IL, USA). Normally distributed data were described by the mean ± standard deviation, while non-normally distributed data were represented by the median (interquartile range). Categorical variables were described by frequency and percentage.

## Results

### Sample characteristics

Between September 2014 and September 2021, a total of 290 EJV line placement attempts were performed. There were 29 cases excluded due to a lack of placement results, leaving 261 cases included in this study. Missing data for height occurred in 24 cases, resulting in 237 cases with complete data. The baseline characteristics of the patients included were described in Table 1. The median age of children at catheter placement was 60.00 months (IQR 76.00; range 1-204 months), with a large proportion of boys (64.80%). The median weight of children at placement was 16.75 kg (IQR 11.18; range 4.10–52.60 kg) with a median height of 113 cm (IQR 39; range 68–168 cm). Of these, the number of infant cases was 48, with a median age of 12.00 months (IQR 2.75; range 1–12 months) and a median weight of 9.05 kg (IQR 2.83; range 4.10–15.50 kg). Data on height were missing for 35.42% (17/48) of infants, resulting in a median height of 75.00 cm (IQR 5; range 68–90 cm). The most common underlying diagnoses requiring catheter placement were leukaemia (n = 146, 55.94%), pulmonary infection (n = 15, 5.36%), and hemophagocytic syndrome (n = 7, 2.68%).

### Placement overview

The anatomical positioning and skin puncturing were 100% successful (261/261). The site of most attempts was the right EJV (n = 169, 64.75%). Failed catheter placements occurred in two infant and four children cases. The leading cause of failure was mechanical: the guidewire could not pass the level of the clavicle (6/261, 2.30%). A total of 20 cases (7.66%) had misplaced tips, which comprised 12 in the internal jugular vein (IJV), five in the subclavian vein (SCV), and three in the brachiocephalic vein (BCV), resulting in a success rate in one visit of 90.04% (235/261) in overall placement. For the 48 infant cases, there were two cases where the catheter could not be advanced and one case where the tip of the catheter was misplaced into the IJV, giving a placement success rate of 93.75% (45/48). The median line duration of use was 19 days (IQR 19; range 2–84 days) with a median length of catheter insertion of 13 cm (IQR 2; range 6-20 cm). Discontinuation of catheters was mainly due to: completion of therapy (n = 166), suspicious infection (n = 16), restoration of normal coagulation (n = 16), death (n = 9), and dislodgement (n = 8). A total of 20 cases with PICCs failed to visit for follow-up, of which 12 patients were discharged to home, and eight were transferred to other centres.

### Complication and management

Complications occurred in 31 cases (11.88%, 31/261), with 21 cases of immediate complications (67.74%, 21/31) (Table 2). The immediate complications included hematoma (n = 1) and catheter malposition (n = 20), while accidental dislodgement was present in seven cases, and infection was seen in three cases (catheter-related bloodstream infection, CRBSI, n = 1; exit-site infection = 2). Up to 71.43% (5/7) of accidental dislodgement occurred in the infant cases. With the addition of one point each of CRBSI, exit-site infection, and catheter malposition, the complication rate in infant cases was 16.67% (8/48). The hematoma dissipated with local compression while the misplaced catheters were removed. Initially, 16 cases were suspected of catheter-related bloodstream infection, all of which were removed and cultured for bacteria and fungi, with only one case cultured for staphylococcus aureus. The cases that developed exit-site infection improved with antimicrobial treatment and were retained for use.

**Table 2:** Complications details (n = 31)

Variable	
Immediate complications, n (%)	**21, (67.74)**
Hematoma, n	1
Catheter malposition, n	20
Delayed complications, n (%)	**10, (32.26)**
Dislodgement, n	7
Infection	
Catheter-related bloodstream infection, n	1
Exit-site infection, n	2

## Discussion

This study reported a 7-year paediatric practice in our department that the experienced nurse used the CVCs to perform the PICC via the EJV approach for children without GA. It has a pleasing success rate in anatomical localisation (100%), punctures (100%) and catheterisation (90.04%), with an acceptable rate of complications (11.88%), and might be an alternative to conventional PICC for paediatric patients with inaccessible access and a high risk of GA.

Hospitalised children require the placement of a peripheral inserted central catheter to receive non-peripherally compatible infusates for life-saving therapies and to facilitate blood tests [17]. However, this procedure is not always a breeze for RNs. On the one hand, the presence of altered venous anatomy from congenital or acquired conditions can pose technical difficulties during the placement [18], especially in large referral centres, where the vascular condition of children on admission may not be promising due to multiple previous placements and severe medical conditions [19]. On the other hand, GA may expose children with respiratory infections and coagulation disorders to a higher risk. The EJV placement may help paediatricians out. Firstly, the EJV is the largest of the superficial jugular veins, and even in children with difficult vascular access, it is still easy to visualise and palpate [20]. Its localisation and puncture are virtually effortless, as in our study, where its success rate is 100%. Secondly, it does not require GA. It is acceptable even if the child is uncooperative or cries and fusses. In fact, crying instead helps in the placement because the EJV would be more visible when the child is crying or fussing. The non-essential requirement for GA makes the risks related to it effectively be avoided to some extent. Our institution also uses it as a temporary alternative to conventional PICC for children with coagulopathy. When patients’ coagulation is normal, this practice will be terminated, and a new peripheral inserted central catheter will be placed in the routine access. Thirdly, this practice does not restrict children’s upper limb movement. The arm’s position significantly influenced the conventional PICC central tip location, moving it an average of 2.2 rib spaces, a maximum of 3.5 ribs. Elbow bending and adduction of the arm caused the central tip to move deeper into the chest [21]. As for our practice, any movement of the child’s upper limbs is permitted. Finally, this practice could be conducted by trained RNs, and the price of a CVC is nearly one-tenth of a peripheral inserted central catheter, which both help in decreasing the overall cost of healthcare [18]. Nonetheless, there are inherent deficiencies in this practice. Firstly, the rate of catheter malposition of the EJV placement might be high. This could be attributed to the anatomical characteristics of the EJV [22]. When the angle of junction of the SCV/IJV and the EJV is small, the catheter might be misplaced into the ipsilateral vein. When the angle is large, it might tend to be misplaced into the contralateral vein, and when the EJV is branched, the length of catheter placement measured from the body surface may not be long enough to reach the desired portion [23]. Secondly, immobilising catheters in children with short and thick necks is challenging. Thirdly, the patient’s physical removal of the catheter becomes easier when the child’s upper limbs are unrestricted. As a result, the risk of accidental dislodgement is higher. Visualisation techniques may solve the problem with vascular anatomy. However, our practice is not under GA and visualisation techniques such as ultrasound are not of assistance in our opinion if the child is uncooperative. More discussion is needed. Better catheter fixation dressings would help with the second flaw, although it would mean more medical expenditure.

In fact, catheterisation via the EJV access using a CVC was not an unfamiliar practice. It was often regarded as centrally inserted central catheterisation and performed by clinicians [24, 25]. However, from our perspective, it should be considered as the PICC which can be performed by RNs, because the EJV is a peripheral vein, although a CVC is used in this practice rather than a peripheral inserted central catheter. In published studies, it has a high success rate in both adults (78–100%) [24, 26, 27] and paediatric populations (> 90%) [12, 28]. The success rate of our study was similar to that of the published (Alshafei’s study [28], n = 252, 91.30%; Tecklenburg’s study [12], n = 50, 90.00%), while the success rate in infants was higher than that in Tecklenburg’s (n = 8,50%) [12]. The sample size might have contributed to the difference; our research included 48 infant cases, which was 6 times more than his [29]. Additionally, the operator’s experience may have influenced the results [30], but this factor should be considered seriously as it cannot be objectively quantified. Compared to conventional PICC [31, 32], the success rate of placement in this study was slightly lower. However, the sample of our study included more than 20% of infant cases, and the success rate in one visit in this study was appreciable compared to studies that had a similar percentage of infant cases (Badheka’study [33], 79.6%; Yu’s study [34], 52.7%). The overall complication rate of EJV placement was 11.88%, with malposition and dislodgement as the two most common complications. The rate of catheter malposition in our study was higher than in Wu’s study [35], Simonetti’study [36], and Ligia’study [37], lower than that in Suzuki’study [32], Yu’s study [34], and Jumani’s study [38], and about the same as in Badheka’s [33]. The considerable variation in the malposition rate may be in part explained by differences in disease types and age distribution of the population studied [33]. The dislodgement rate in our research was higher than in published studies [32, 39, 40], which was related to the inherent shortcomings of our practice. Up to 71.43% of accidental dislodgement occurred in the infant cases, which, in line with our analysis, children with short, thick necks were at higher risk of dislodgement. Exit-site infection was reported in two cases, and CRBSI was recorded in only one case, as in other studies [41, 42]. In six cases, the EJV placement failed because of the inability to advance the guidewire. This was our initial protocol that ended catheterisation when it was unable to advance the guidewire and regarded it as a failure case. We later found that an attempt to advance the catheter in this situation might be helpful, but we do not recommend forcible delivery if it cannot be advanced either. Overall, the success and complication rates of this practice are acceptable.

### Limitations

There were several limitations in this study. Firstly, it was a retrospective observational study. The data listed in the medical records was recorded by different RNs at different times, and there might be certain deficiencies in the completeness of the data. Secondly, this was a single-centre study, and its feasibility and safety need to be verified in a prospective or multicentre study. Thirdly, there were inherent deficiencies in this practice. Further discussion and appropriate assistance are needed.

## Conclusion

In conclusion, paediatric PICC via the EJV approach using a CVC without GA is a feasible and safe practice with acceptable success and complication rates, and low costs. It might be an effective alternative for obtaining central vascular access for paediatric patients. However, more research is needed to determine if this practice can be broadly applicable and how to improve the inherent deficiencies.

### Electronic supplementary material

Below is the link to the electronic supplementary material.

Supplementary Material 1

## Data Availability

Restrictions apply to the availability of these data. Data were obtained from HIS and are available from the author team with the permission of Wang-mei Zhou and Ping Zhang.

## Abbreviations

PICC: Peripherally insertion central catheterization
GA: General anaesthesia
EJV: External jugular vein
CVC: Central venous catheter
RN: Registered nurse
IJV: Internal jugular vein
SCV: Subclavian vein
BCV: Brachiocephalic vein
