# Supplementary material for: Cannulation via the external jugular vein——An alternative to conventional peripherally inserted central catheterisation for paediatric patients
Source: BMC Pediatr. 2023 Nov 18;23:579. doi: 10.1186/s12887-023-04403-5 (PMC10657000; doi:10.1186/s12887-023-04403-5)
Supplement: Supplementary file 1 — Supplementary Material 1 [file 12887_2023_4403_MOESM1_ESM.docx]

**Appendix Table 1 - Definitions of complications**

| **Complications** | **Definition** |
| --- | --- |
| **Immediate** |  |
| Bleeding/ hematoma | The site of insertion still bleeding over 72 h after PICC insertion or serious bleeding needs blood transfusion/ Local hematoma |
| Arterial puncture | Inadvertent needle puncture of an associated artery |
| Arrhythmia | Ventricular dysrhythmias and bundle branch block |
| Air embolism | The sudden onset of dyspnea, gasping, continued coughing, breathlessness, chest pain, hypotension, tachyarrhythmias, wheezing, tachyp- nea, altered mental status, altered speech, changes in facial appearance, numbness, or paralysis as clinical events from air emboli produce cardiopulmonary and neurological signs and symptoms. |
| Catheter malposition | Catheter tip was not terminated in the superior vena cava or right atrium. |
| Pneumothorax | The entry of air into the pleural space as detected by chest radiography |
| Nerve injury | Temporary or permanent nerve injury during insertion and dwell |
| **Delayed** |  |
| Infection |  |
| Suspected infection involving PICC | Suspicion by treating clinician for infection that involved PICC. Patient’s symptoms may have included fever, increased white blood cell count, or increased inflammatory markers (CRP, ESR) without other source. |
| Catheter-related bloodstream infection | Isolation of the same micro-organism in peripheral blood and PICC cultures; threefold difference in paired quantitative cultures of blood samples drawn from PICC an peripheral vein; or two hours earlier culture positivity in blood collected from PICC than in blood from peripheral vein. |
| Exit site infection | The presence or purulent discharge with erythema and/or tenderness close to the catheter exit site. |
| Systemic infections | Fever, chills, malaise, or hemodynamic instability as signs of bacteremia and sepsis, or with endocarditis or septic emboli. |
| Catheter-associated deep vein thrombosis, CA-DVT | CA-DVT is often clinically silent and does not produce overt signs and symptoms. Clinical signs and symptoms are related to obstruction of venous blood flow and may include, but are not limited to, pain/edema/erythema in the extremity, shoulder, neck, or chest and engorged peripheral veins of the extremity. Diagnose and confirm CA-DVT using color-flow Doppler ultrasound by the presence of at least 2 of the following: noncompressability of the vein, abnormal color Doppler vein pattern, and/or IV filling defect. |
| Venous stenosis | Venous stenosis with or without symptoms confirmed by Duplex ultrasound ultrasound. |
| Catheter malfunction |  |
| Dislodgement | Accidental or unplanned physical removal of the catheter by either the patient or the nursing staff. |
| Breakage | Tears, splits or breaks along the length of the catheter. |
| Occlusions | Inability to withdraw blood or sluggish blood return; Sluggish flow; resistance or inability to flush lumen; inability to infuse fluid; Frequent occlusion alarms on electronic infusion pump; Swelling/leaking at infusion site. |
| Leakage | Leakage of infusate from the insertion site, without other signs or symptoms of infiltration. |
| Infiltration/Extravasation | Swelling, pain, whitening or reddening of the skin in the area near the puncture. |
| Phlebitis | Pain/tenderness, erythema, swelling, purulence, or palpable venous cord. |
| Others | Any unusual occurrence that does not fit into any of the above specified complications, such as myocardial perforation. |
